# Photocatalytic Degradation of Profenofos Using ZnO Nanoparticles Biosynthesized with Aqueous Grape Seed Extract

**DOI:** 10.3390/molecules31132221

**Published:** 2026-06-24

**Authors:** Elvis Gilmar Gonzales-Condori, Rocio Janeth Jove-Roman, Alfredo Quispe-Mamani, Gerson Márquez, Jeaneth Medina-Pérez, José Miguel Carpio-Carpio, Luis Lipa-Mamani, José A. Villanueva-Salas

**Affiliations:** 1 Grupo de Investigación en Biotecnología y Ciencia de los Alimentos (GIBYCA), Universidad Tecnológica del Perú (UTP), Av. Tacna y Arica 160, Arequipa 04001, Peru; 2 Escuela Profesional de Farmacia y Bioquímica, Universidad Católica de Santa María (UCSM), Urb. San José s/n Umacollo, Arequipa 04013, Peru; 3 Facultad de Agronomía, Universidad Nacional de San Agustín de Arequipa, Arequipa 04001, Peru

**Keywords:** ZnO nanoparticles, degradation, grape seeds, aqueous extract, water

## Abstract

The use of organophosphate pesticides, such as profenofos, is a pressing environmental concern due to their persistence and toxicity to non-target organisms. For this reason, developing alternatives to remove them from the environment is crucial. The objective of this study was to biosynthesize ZnO nanoparticles (ZnO NPs) using an aqueous extract of Negra Criolla grape seeds for their application in the degradation of profenofos in synthetic solutions. The biosynthesized ZnO NPs had an average size of 52 ± 2 nm and a maximum absorption at 375 nm, characteristic of the surface plasmon resonance of ZnO NPs. The ATR-FTIR spectra showed peaks characteristic of ZnO NPs. The 2^2^ × 3 factorial design showed that the optimal values for pH and ZnO NPs concentration are 5 and 3 g/L, respectively, achieving a primary degradation of profenofos (20 mg/L) of 59 ± 2% after 120 min of UV irradiation. In conclusion, it was demonstrated that ZnO NPs biosynthesized using an aqueous extract of Negra Criolla grape seeds exhibit photocatalytic activity for the degradation of profenofos; however, further studies are needed to evaluate their application in actual contaminated water.

## 1. Introduction

Population growth has created high demand for agricultural products to feed approximately 7 billion people worldwide [1]. For this reason, pesticides are commonly used to prevent, destroy, repel, or control pathogens and pests that could potentially harm crops [2]. Nowadays, environmental pollution caused by agrochemicals is a global problem, with pesticides being the main substances used in agro-industrial activities for pest control [3]. Agriculture is one of the main economic activities in Peru [4]. Organophosphorus (OPPs) pesticides are commonly used in agriculture [5]. OPPs irreversibly bind to acetylcholinesterase, making them effective at triggering cholinergic symptoms that can kill insects and even humans, which makes these compounds effective for pest control; however, humans and non-target organisms may be affected by the residual effects of these OPPs, which can leach into deep soil layers and reach groundwater, or run off into irrigation canals and ultimately into rivers [6]. Compounding this problem is the use of contaminated water for human consumption, as some communities in Arequipa lack access to potable water and rely on groundwater or river water that has been treated in ways that do not remove toxic molecules such as OPPs. OPPs account for approximately 28% of insecticides sold worldwide [7]. It is well known that OPPs have contributed significantly to agricultural activities by reducing the risk of crop losses due to their effectiveness against pests; however, the problem lies in the fact that these OPPs persist and bioaccumulate in the environment, posing a potential risk to mammals and non-target organisms in ecosystems adjacent to application areas [8,9].

Among the most commonly used OPPs is profenofos (PF), known for its effectiveness in controlling pests resistant to other OPPs, such as chlorpyrifos [10]. PF is widely and indiscriminately used in agricultural [11,12,13] and horticultural crops to control flying, crawling, chewing, and sucking insects [14]. For years, studies and process applications have been developed to accelerate the removal of OPPs from soil to prevent these toxic substances from entering the water. Among the options for degrading OPPs is the isolation of native soil microorganisms; this appears to be a highly promising technology for removing OPPs [1] such as chlorpyrifos [15,16] and PF [9,11]; likewise, photocatalytic [17,18] and microwave [19] processes were developed and also proved effective. In photocatalytic processes, various semiconductors are used to generate hydroxyl radicals that degrade organic matter; among these semiconductors are nanoparticles that can be synthesized using physical and chemical methods, as well as through biosynthesis or eco-synthesis [20]. Green synthesis enables the development of safer nanomaterials and addresses safety, health, and environmental concerns. Furthermore, these biogenic particles possess unique properties that make them suitable for applications in medicine, agriculture, bioengineering, and bioremediation [21]. The eco-friendly synthesis method for producing ZnO nanoparticles (ZnO NPs) employs natural waste materials that act as reducing agents and stabilizers [22]. For these reasons, ZnO NPs are entirely feasible for laboratory development, given their advantages, such as energy efficiency, low toxicity, high performance, high cost-effectiveness, environmental friendliness, and wide availability [23]. Some plant species contain reducing compounds, such as grape seeds, which are essential for the synthesis of nanoparticles [24]. Furthermore, the use of agro-industrial waste, such as grape seeds, plays a key role in the circular economy and sustainable development [25]. For this reason, this study aims to demonstrate that grape seeds are a source of reducing agents for the synthesis of ZnO nanoparticles, employing grape seed extract as the reducing agent, and subsequently applying these nanoparticles to remediate water contaminated with the organophosphate pesticide profenofos using synthetic solutions.

## 2. Results and Discussion

### 2.1. Characterization

#### 2.1.1. Characterization by ATR-FTIR of GS and GSAE

**Figure 1:**
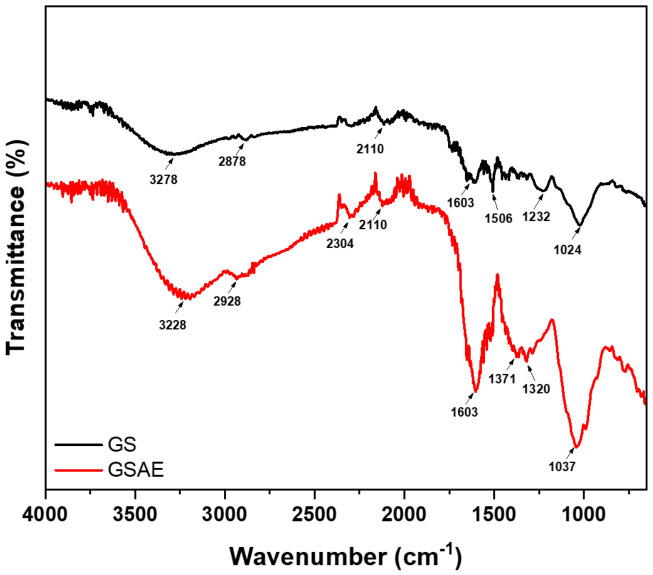
ATR-FTIR spectra of grape seeds (GS) and aqueous grape seed extract (GSAE).

Figure 1 shows the FTIR spectra obtained via ATR-FTIR of pressed grape seeds (GS) (oil-free) and grape seed aqueous extract 5% (GSAE-5%). Given that both matrices represent complex organic mixtures containing a wide variety of secondary metabolites (such as polyphenols, flavonoids, fatty acids, and tannins), their infrared profiles exhibit inherently broad bands that reflect the overlapping signals of multiple functional groups rather than single isolated compounds. Consequently, these spectra are presented primarily as characteristic qualitative fingerprints of GS, GSAE-5%, and the extracting agent.

The black line spectrum corresponds to the pressed and pulverized GS, where a peak at 3228 cm^−1^, characteristic of the stretching vibrations of the -OH groups, is observed. These signals may be associated with the phenolic compound content in the seeds [26]. A characteristic peak corresponding to the stretching vibrations of C-H groups appears at 2878 cm^−1^. A peak corresponding to nitrogen-containing groups appears at 2110 cm^−1^. At 1603 and 1506 cm^−1^, a characteristic peak corresponding to the stretching vibrations of the C=C groups is observed; at 1232 cm^−1^, C-H bending vibrations are present; finally, a peak at 1024 cm^−1^ corresponding to the wagging vibrations of -CH_3_ is observed. The spectra of the 5% GSAE are shown in red, and it can be observed that the same signals from the functional groups found in the pressed GS are present; however, these signals are more intense, specifically at 3228 cm^−1^, which could correspond to flavonoids, alkaloids, carboxylic acids, or polyphenols [27] that were extracted with water.

#### 2.1.2. Characterization of ZnO NPs

**Figure 2:**
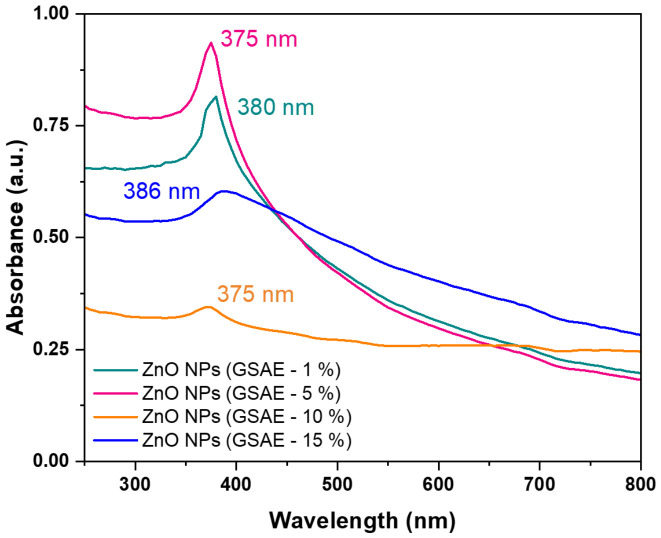
Spectrophotometric scans of ZnO nanoparticles biosynthesized using grape seed aqueous extracts (GSAE).

Figure 2 shows the UV/vis spectra of the four ZnO NPs suspensions biosynthesized using 1%, 5%, 10%, and 15% of grape seed aqueous extracts (GSAE). These spectra correspond to the surface plasmon resonance phenomenon. The ZnO NPs biosynthesized using 1% GSAE exhibited a spectrum with a maximum peak at 380 nm (green line); likewise, the ZnO NPs biosynthesized with 5% GSAE developed a peak at 375 nm (pink line); similarly, the ZnO NPs obtained with 10% GSAE (orange line). Finally, the ZnO NPs obtained using GSAE-15% exhibited a peak at 386 nm (blue line). In another study, ZnO NPs were biosynthesized using an extract of *Cymbopogon proximus*, yielding a maximum absorption peak at 375 nm [28], and using an extract of *Amaranthus dubius*, ZnO NPs were biosynthesized with an absorption peak at 377 nm [29]. These absorption peaks demonstrate the formation of ZnO NPs from the zinc salts used in the synthesis [30]. For the degradation experiments, ZnO NPs biosynthesized using 5% GSAE were used because they have a sharper absorption peak and higher absorption [31].

The optical bandgap (*E*g) was determined from the absorption spectra shown in Figures S1–S4 of the Supplementary Material using the Tauc method [32,33]. The bandgap of the ZnO NPs biosynthesized with 1%, 5%, and 10% GSAE was 2.84 eV (Figure S1), 2.92 eV (Figure S2), and 2.58 eV (Figure S3), respectively. These results show a smaller-than-expected bandgap for bulk ZnO (*E*g = 3.37 eV). These values are similar to those reported in other studies in which ZnO NPs were biosynthesized, suggesting that this may be due to the nanoparticles’ small size or to the use of plant extracts as reducing agents [34]. A higher concentration of grape seed extract during synthesis would result in a higher density of oxygen- and carbon-rich organic compounds (such as polyphenols and flavonoids) bound to the zinc precursors. Following thermal calcination, these surface organic residues would introduce localized structural defects, which could effectively reduce the sample’s optical bandgap by 10% GSAE. Figure S4 shows the Tauc plot of the ZnO nanoparticles synthesized using a 15% aqueous grape seed extract; however, the bandgap value could not be calculated due to matrix (extract) interference.

**Figure 3:**
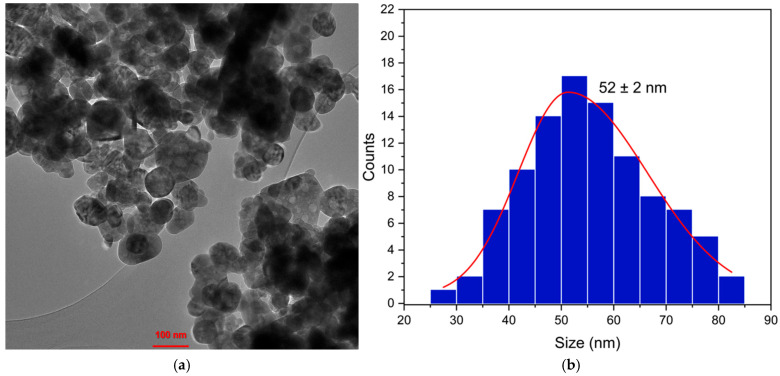
(**a**) Transmission electron microscope (TEM) micrograph of ZnO nanoparticles and (**b**) histogram for nanoparticle counting.

Figure 3a shows a TEM micrograph of the biosynthesized ZnO NPs, revealing spherical and irregularly shaped nanostructures. On the other hand, Figure 3b presents the histogram of biosynthesized ZnO NP diameters, indicating an average size of 52 ± 2 nm.

On the other hand, Table 1 presents a comparison of various biosynthesized ZnO NPs with other aqueous extracts reported in previous studies, showing the range of particle sizes that can be achieved depending on the extract and calcination temperature.

**Figure 4:**
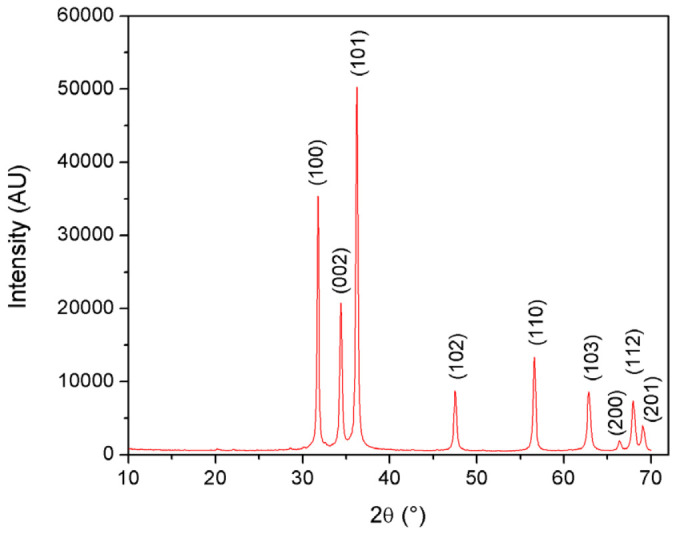
X-ray diffraction pattern of the ZnO nanoparticles.

Figure 4 shows the XRD pattern of the synthesized ZnO nanoparticles, displaying several intense and well-defined diffraction maxima. All observed peaks were indexed to the hexagonal wurtzite phase of ZnO, with space group P6_3_mc (No. 186), in agreement with the standard PDF card No. 36-1451. The indexed diffraction maxima are shown in Figure 4.

Le Bail refinement provided unit cell parameters of a = b = 3.2513(2) Å and c = 5.2076(5) Å, in good agreement with those reported for wurtzite ZnO in PDF card No. 36-1451 (a = b = 3.2498 Å and c = 5.2066 Å) and in previous studies on zinc oxide [40,41]. Based on the unit cell volume (V = 47.67 Å^3^), the X-ray density was calculated as 5.67 g cm^–3^, which is consistent with the literature values for ZnO, typically ranging from 5.00 to 5.68 g cm^–3^ [42,43]. The average crystallite size, estimated from the position and full width at half-maximum (FWHM) of the most intense diffraction peak, assigned to the (101) plane, using the Scherrer equation [44] with a shape factor (K) of 0.9, was approximately 34 nm.

**Figure 5:**
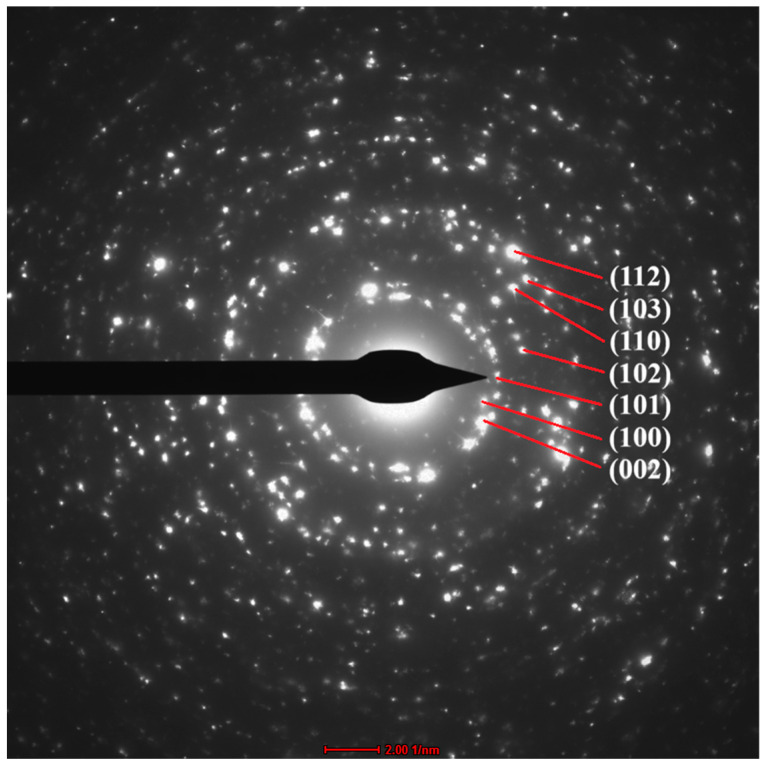
Selected-area electron diffraction pattern of the ZnO nanoparticles.

Figure 5 shows the selected-area electron diffraction pattern of the synthesized ZnO nanoparticles. The pattern displays diffraction maxima arranged as spotty rings, characteristic of a polycrystalline material composed of highly crystalline grains or particles. Indexing of these maxima confirmed that the nanoparticles crystallized in the hexagonal wurtzite structure of zincite (ZnO), thereby supporting the structural results obtained by X-ray diffraction.

**Figure 6:**
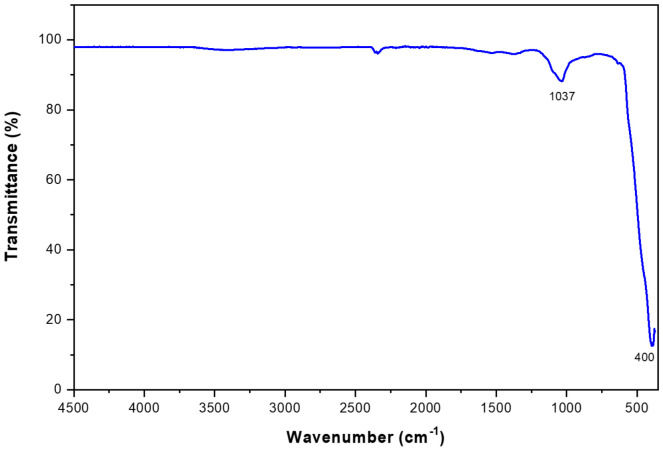
ATR-FTIR spectra of ZnO nanoparticles biosynthesized.

Figure 6 shows the ATR-FTIR spectrum of the biosynthesized ZnO NPs, in which a small peak is observed at 1037 cm^−1^, while a peak at 400 cm^−1^ is also observed, which could correspond to the ZnO NPs. These signals correspond to the Zn-O stretching mode, which appears between 400 and 850 cm^−1^ and is characteristic of the hexagonal crystal structure of ZnO [31,45]. This is confirmed by another study, which indicates these signals correspond to the formation of ZnO NPs [27].

**Figure 7:**
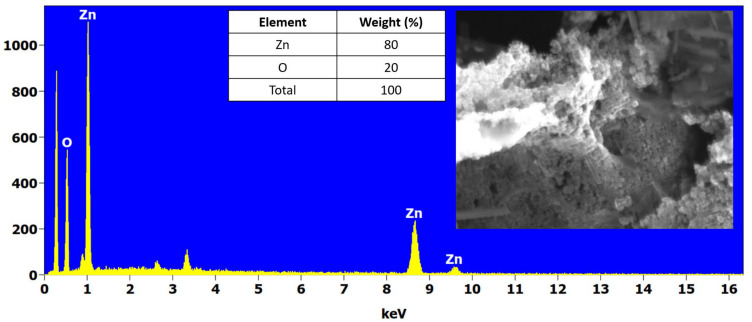
SEM-EDS analysis of ZnO nanoparticles biosynthesized.

Figure 7 shows the surface morphological and elemental profile obtained by SEM-EDS analysis of the biosynthesized ZnO NPs. A substantial state of nanoparticle agglomeration is clearly visible in the low-magnification SEM micrograph, which contrasts with the high-resolution individual nanostructures isolated via TEM in Figure 3a. Furthermore, the EDS analysis revealed the presence of primarily Zn and O, with weight percentages of 80% and 20%, respectively.

**Figure 8:**
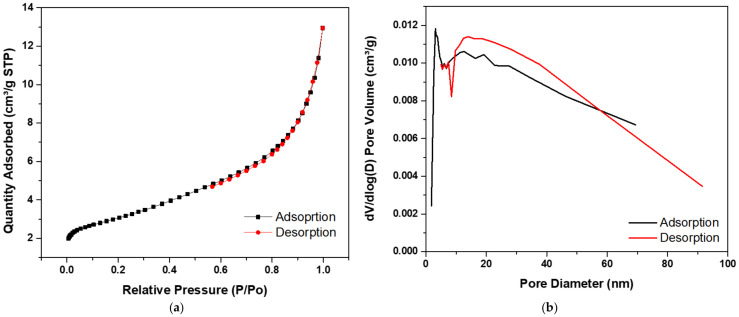
(**a**) BET Adsorption/Desorption isotherm and (**b**) BJH plot.

The textural properties and porosity structure of the biosynthesized ZnO NPs were analyzed using nitrogen physisorption. The results corresponding to the adsorption–desorption isotherm, pore size distribution, and calculated quantitative parameters are presented in Figure 8a, Figure 8b, and Table 2, respectively. As shown in Figure 8a, the physisorption profile of the ZnO NPs follows a Type IV isotherm, characteristic of the presence of predominantly mesoporous materials. It is noteworthy that the adsorption and desorption curves exhibit an extremely narrow hysteresis loop in the relative pressure (P/P0) range of 0.5 to 0.9. The absence of pronounced hysteresis suggests that the mesoporosity corresponds to interparticle porosity resulting from the agglomeration of the nanoparticles.

Furthermore, Figure 8b shows the pore-size distribution curve calculated using the BJH (Barrett–Joyner–Halenda) analysis. The graph shows a detailed heterogeneous distribution ranging from 2 nm to values greater than 60 nm. The first, very sharp, and narrow peak is identified, centered between 3 and 4 nm, indicating the presence of a uniform population of very small mesopores, likely located in the closest-contact zones or at the junctions of the nanoparticle network. The curve shows a broad plateau between 10 and 40 nm, which could indicate a polydisperse, random nature of the interstitial pores, due to the packing of agglomerates of different sizes. The average pore diameter is 28 nm, which could confirm the formal classification of the material as a mesoporous system.

### 2.2. Degradation of Profenofos Using ZnO Nanoparticles

#### 2.2.1. Optimization

Table 3 presents the results of profenofos degradation efficiency, expressed as a percentage, based on the twelve experiments of the 2^2^ × 3 factorial design, which considered the factors of pH (4 and 5) and ZnO NPs dosage (1 and 3 g/L).

**Figure 9:**
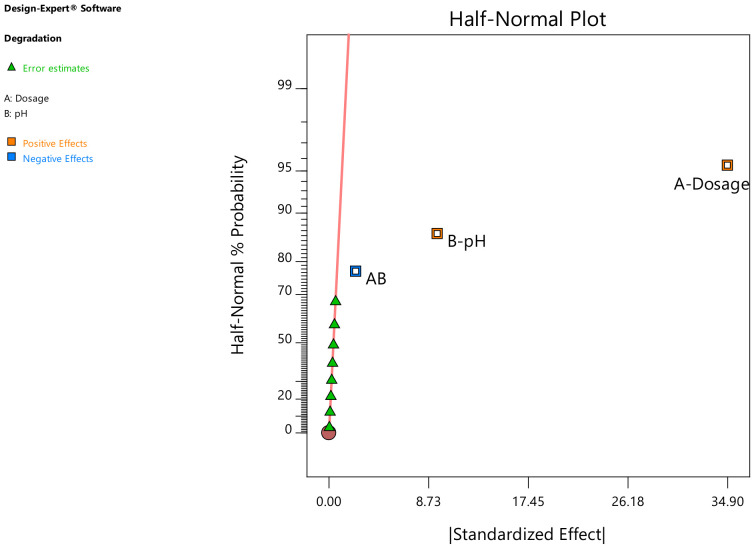
Half-normal plot for evaluating the factors studied in factorial design 2^2^ × 3.

According to the half-normal plot (Figure 9), the dosage of ZnO NPs is the factor with the greatest positive influence on the degradation process; similarly, pH also has a positive influence. In contrast, the interaction between dosage and pH has a negative effect on the degradation process.

Table 4 presents the analysis of variance for the 2^2^ × 3 factorial design. The *p*-values are less than 0.05, indicating that the model and the factors studied have a significant influence on profenofos degradation.

Table 5 shows that the value R^2^-predicted for the model is 0.9951. This value is reasonably consistent with the R^2^-adjusted value (0.9972), which is adequate; furthermore, the precision was found to be greater than 4 (value = 76.206), indicating an adequate signal, and allowing the model to be used for optimization and theoretical prediction of degradation percentages.

The mathematical model is presented in Equation 1 below. This equation can be used to make theoretical predictions of profenofos degradation, considering the ZnO NPs dosage and pH.

(1) Degradation (%)=−60.192+28.076(Dosage)+14.219(pH)−2.361(Dosage)(pH)

**Figure 10:**
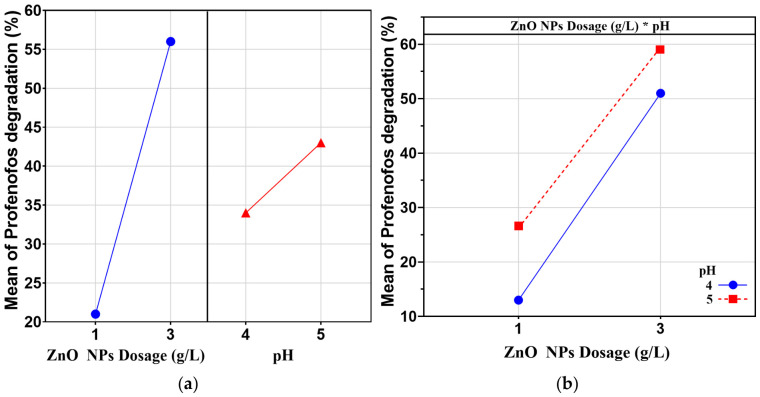
(**a**) Main effects plot and (**b**) interaction plot for the degradation of profenofos as a function of ZnO NPs dosage and pH (*, interaction between ZnO NPs dosage and pH).

Figure 10a shows the main effects plot for the degradation of profenofos with respect to the factors of ZnO NP dosage and pH. It is observed that the highest degradation efficiency is achieved at a dosage of 3 g/L, and at pH = 5. Figure 10b shows the interaction plot for the degradation of profenofos, where pH has a positive effect at both low and high ZnO NPs dosage. This indicates a positive interaction between ZnO NPs dosage and pH with respect to profenofos degradation.

**Figure 11:**
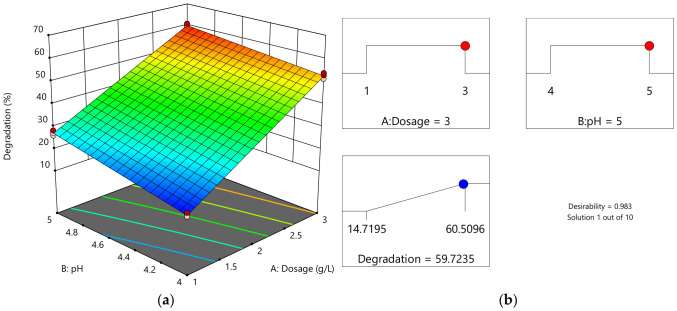
(**a**) Surface plot and (**b**) ramp plot for the optimization of profenofos degradation as a function of ZnO NPs dosage and pH.

Figure 11a shows the surface plot of profenofos degradation, which indicates that degradation efficiency increases with higher dosages. Similarly, degradation efficiency also increases with higher pH. Furthermore, the greatest degradation occurs at higher ZnO NPs dosage and higher pH.

The degradation process of profenofos was optimized with the objective of maximizing the percentage of profenofos degradation; regarding the dosage values of ZnO NPs and pH, which were suggested by the optimization ramp tool in Design Expert software v.11 (Figure 11b), resulting in a 59.72% degradation efficiency of profenofos using an optimal ZnO NPs dosage of 3 g/L and an optimal pH of 5, with a desirability of 0.983.

#### 2.2.2. Kinetics

**Figure 12:**
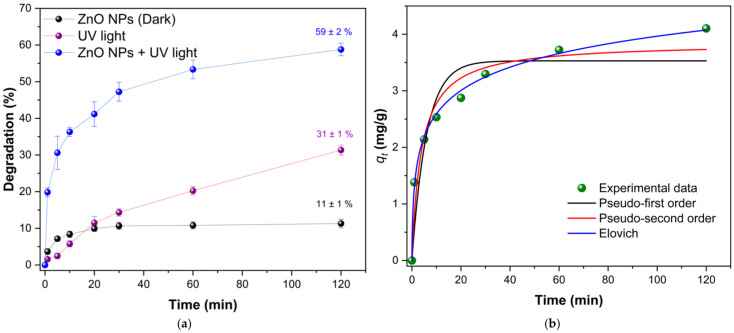
(**a**) Comparison of profenofos degradation via dark adsorption (ZnO Dark), direct photolysis (UV light), and photocatalysis (ZnO + UV light). (**b**) Graph of pseudo first order, pseudo second order, and Elovich kinetic model fits for profenofos degradation.

Degradation experiments of profenofos (initial concentration of 20 mg/L) were conducted at optimal ZnO NPs dosages and pH levels. Monitoring times were at 0, 1, 5, 10, 20, 30, 60, and 120 min. In addition, control experiments were conducted involving direct photolysis (UV light without ZnO NPs) and adsorption in the dark (ZnO NPs without light). Figure 12a shows the degradation efficiencies as a function of time. Under dark conditions (ZnO NPs Dark), a degradation of profenofos of 11 ± 1% was registered, reaching equilibrium after 20 min of stirring. This would indicate that profenofos molecules have an affinity for the mesoporous surface of the ZnO NPs. On the other hand, direct UV photolysis (UV light) produced a primary degradation of 31 ± 1% at 120 min, demonstrating that profenofos would undergo partial direct photolytic degradation under the studied UV radiation. It is important to note that the simultaneous presence of ZnO NPs and UV light (ZnO NPs + UV light) triggered a synergistic effect, maximizing primary degradation efficiency to 59 ± 2%. This notable improvement could be attributed to the continuous generation of reactive oxygen species on the surface of the ZnO NPs, which accelerates the decomposition of profenofos. This experimental degradation value (59 ± 2%) corresponds exactly to the average of three independent experiments conducted exclusively under optimized conditions, demonstrating the high precision of the advanced oxidation process under optimal parameters. Three distinct batches of ZnO nanoparticles were synthesized independently using three different aqueous grape seed extracts prepared prior to each synthesis. The low standard deviation demonstrates that the phytochemical matrix of the agro-industrial waste from the Negra Criolla variety provides a reliable and robust framework for the stable replication of the catalyst.

Figure 12b shows the fit of the adsorption capacities as a function of time to the pseudo-first order, pseudo-second order, and Elovich nonlinear kinetic models. Graphically, the pseudo-second-order and Elovich models provide a better fit than the pseudo-first-order model.

Table 6 presents the parameter values for the kinetic models. The pseudo-second order model has an R^2^ of 0.9441, which is higher than the pseudo-first order model (R^2^ = 0.8775). Furthermore, the Elovich kinetic model shows an excellent fit (R^2^ = 0.9967), which would indicate a highly heterogeneous surface; this is consistent with the structural and biogenic nature of the ZnO biosynthesized in this study. As established by XRD, TEM, and BET characterizations, the nanoparticles exhibit a mesoporous structure formed by compact aggregates, which gives rise to a wide variety of surface defects, interstitial bonds, and uncompensated crystal edges. It was also observed that the adsorption capacity of profenofos on ZnO NPs at equilibrium is 3.851 mg/g, with a rate of 0.067 g.mg^−1^min^−1^.

Table 7 shows the degradation percentages of profenofos obtained using different catalysts, supporting the use of these materials for the removal of organophosphate pesticides such as profenofos.

**Figure 13:**
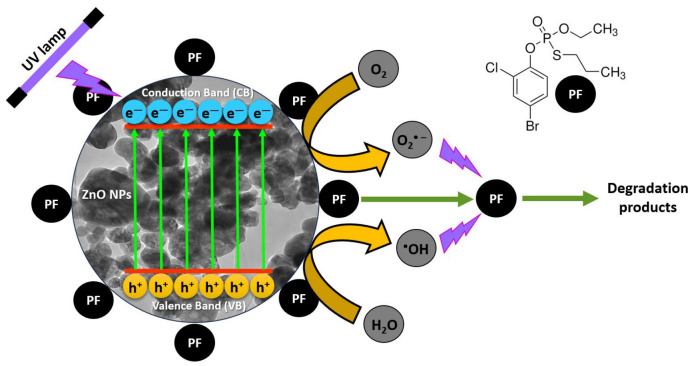
Proposed mechanism of profenofos degradation.

Figure 13 shows a schematic diagram of the degradation mechanism of profenofos using ZnO NPs biosynthesized from an aqueous extract of defatted grape seeds. This mechanism aligns with fundamental principles of semiconductor photocatalysis documented in the literature [48]. The process is initiated when photons from the UV radiation (30 W UV lamp) source (hν) strike the catalyst with an energy equal to or greater than the optical bandgap of the ZnO NPs (2.92 eV), promoting the separation and migration of charge carriers (electrons ($e_{CB}^{-}$) and holes ($h_{VB}^{+}$)) (Equation (2)). The $e_{CB}^{-}$ electrons interact with adsorbed oxygen molecules to generate superoxide anion radicals ($•O_{2}^{-}$) (Equation (3)), while the $h_{VB}^{+}$ holes interact with water molecules or hydroxyl ions to generate highly reactive hydroxyl radicals (•OH) (Equation (4)). Crucially, the subsequent degradation of the organophosphate pesticide does not take place exclusively at the ZnO NPs surface; instead, it proceeds via two simultaneous pathways (Equation (5)):

(2) $$\mathrm{ZnONPs}\overset{hv}{\to }\mathrm{ZnONPs}\;(e_{CB}^{-})+\mathrm{ZnONPs}\;(h_{VB}^{+})$$

(3) $$\mathrm{ZnONPs}\;(e_{CB}^{-})+O_{2}\to \mathrm{ZnONPs}\;+•O_{2}^{-}$$

(4) $$\mathrm{ZnONPs}\;(h_{VB}^{+})+H_{2}O\to +\mathrm{ZnONPs}\;+{\;H}^{+}+•\mathrm{OH}$$

(5) $$•\mathrm{OH}+•O_{2}^{-}+\mathrm{PF}\to \mathrm{Degradation}\;\mathrm{products}$$

### 2.3. Limitations

Although the biosynthesized ZnO nanoparticles demonstrated competitive performance in the primary degradation of profenofos in synthetic solutions, their long-term structural stability and reusability over multiple reaction cycles were not experimentally evaluated at this stage of the study. This aspect constitutes a recognized technical limitation, as zinc oxide semiconductors are known to undergo partial photocorrosion under prolonged ultraviolet radiation in aqueous environments.

On the other hand, the chromatographic methodology used only allowed for monitoring the primary degradation of the profenofos molecule. This is a limitation of the present study, as total mineralization into inorganic species was not quantified through total organic carbon analysis. However, according to the literature on the photocatalytic degradation of organophosphate pesticides, the initial attack by the generated reactive species targets the P=O and P=S groups of the pesticide, leading to its decomposition into phosphate compounds [49]. As a result of this primary degradation of profenofos’s chemical structure, the organophosphate would not bind to the enzyme acetylcholinesterase (AChE), which is an important step in terms of toxicity to non-target organisms.

To bridge the gap between controlled laboratory conditions and real-world applications, it is essential to consider the potential impact of the aqueous matrix composition on photocatalytic performance. Although biologically derived ZnO NPs achieved competitive primary degradation in synthetic solutions, transferring this system to an actual contaminated surface or groundwater introduces complex chemical species, such as natural organic matter and ubiquitous inorganic anions like chlorides, sulfates, and bicarbonates. These matrix components typically hinder photocatalytic kinetics through two main mechanisms: competitive occupation of active sites and radical scavenging [18].

## 3. Materials and Methods

### 3.1. Reagents and Equipment

For this study, HPLC-grade acetonitrile from Merck (Darmstadt, Germany) was used. Profenofos PESTANAL and zinc acetate were obtained from Sigma Aldrich (St. Louis, MO, USA). The 10% Tween was obtained from Bio-Rad (Hercules, CA, USA). Ultrapure water was used in all experiments. The surface plasmon resonance phenomenon was analyzed using the Agilent Technologies Cary 60 UV/Vis Spectrophotometer (Santa Clara, CA, USA). Characterization was performed using the Agilent Cary 630 Attenuated Total Reflectance (ATR)-Fourier Transform Infrared (FTIR) spectrometer. Profenofos quantifications were performed on the Hitachi Primaide high-resolution liquid chromatograph (HPLC, Tokyo, Japan). An RP-18 column (260 × 4 mm) was used. Calcination was performed in a Thermo Scientific Thermolyne muffle furnace, model FB1410M (Waltham, MA, USA). Microphotographs of the nanoparticles were taken using a Thermo Scientific Talos F200i transmission electron microscope.

### 3.2. Seed Collection

In this study, grape seeds of the Negra Criolla variety were used. The seeds, which had been defatted by pressing, were obtained from a company specializing in the production of wine and pisco in the district of Vitor, Arequipa (−16.4717188, −71.9393714). The pressed grape seeds were dried at 40 °C for 24 h and then pulverized in a blade mill. The pulverized seeds were dried a second time at 40 °C for 24 h.

### 3.3. Obtaining the Extract

To determine the extract to be used in the synthesis of nanoparticles, four aqueous extracts were prepared at concentrations of 1%, 5%, 10%, and 15%. To do this, 0.5, 2.5, 5, and 7.5 g of the powder were weighed into reflux flasks, and 50 mL of ultrapure water were added. The cooling system was then connected, and the mixture was boiled for 5 min. The extracts were allowed to cool and were then filtered. The aqueous extracts were used for the synthesis of nanoparticles.

### 3.4. Synthesis of Nanoparticles

**Figure 14:**
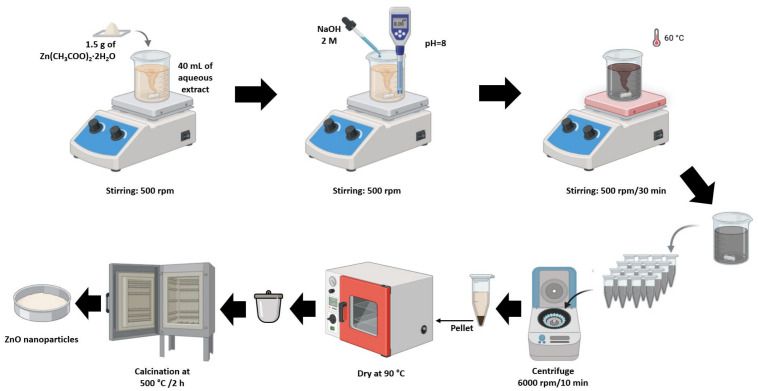
Process for synthesis of ZnO nanoparticles.

The synthesis process for nanoparticles of zinc oxide (ZnO NPs) was adapted from the study conducted by Faisal et al. [50]. The procedure is shown in Figure 14. A total of 2.4 g of zinc acetate dihydrate was added to 40 mL of aqueous extract, followed by the addition of drops of 2 M NaOH until a pH of 8 was reached; the mixture was then stirred at 60 °C on a heated magnetic stirrer for 30 min. It was then allowed to cool and centrifuged at 6000 rpm for 10 min. The supernatant was discarded, and the pellet was washed with ultrapure water and centrifuged three times. It was then dried in an oven at 90 °C. The dry pellet material was calcined for 2 h at 500 °C in a muffle furnace. A white powder was obtained, corresponding to the ZnO NPs.

### 3.5. Characterization of Nanoparticles

The characterization of the biosynthesized ZnO NPs was performed using UV/Vis spectrophotometry [51]. The procedure involved weighing 0.01 g of ZnO NPs into a 10 mL volumetric flask, suspending the nanoparticles in absolute ethanol, and placing it in an ultrasonic bath for 5 min. Subsequently, spectrophotometric scans were performed from 250 to 800 nm. The appearance of a peak of maximum absorption in the scan spectrum between 370 and 380 nm indicates the formation of ZnO NPs, as this is characteristic of the surface plasmon resonance of ZnO NPs [28,29]. In addition, the ZnO NPs, the pressed grape seeds, and the aqueous extract (previously dried in a water bath at 40 °C) were characterized using ATR-FTIR. Finally, characterization was performed using TEM to obtain biogenic nanoparticle micrographs, which were processed in ImageJ software 2.9.0. to calculate the diameters of each nanoparticle. The data were then imported into OriginPro 2026 software to generate a histogram plot showing the average size ± standard deviation.

To analyze the crystalline structure of the synthesized nanoparticles, X-ray diffraction (XRD) measurements were performed using a Rigaku Ultima IV powder diffractometer equipped with Cu Kα radiation (Akishima, Japan, λ = 1.5406 Å). The XRD pattern was recorded over the 2θ range of 10–70°, with a step size of 0.05° and a scan rate of 0.5° min^−1^. The crystalline phases present in the synthesized material were identified using QualX software 8.40 by comparing the experimental pattern with the Powder Diffraction File (PDF-2) database of the International Centre for Diffraction Data (ICDD). The crystallographic parameters were determined through Le Bail refinement using FullProf Suite second version. The theoretical density of the synthesized material, also referred to as the X-ray density (*ρ*_x-ray_), was calculated from the lattice parameters according to *ρ*_x-ray_ = (*Z*·*M*)/(*V·N*_A_), where *Z* represents the number of formula units per unit cell, *M* is the molar mass of the compound, *V* is the unit cell volume, and *N*_A_ is Avogadro’s constant.

Selected area electron diffraction (SAED) patterns were recorded using a Thermo Scientific Talos F200i transmission electron microscope. The SAED patterns were processed in DigitalMicrograph 3.7.1 by measuring the radii of the diffraction rings associated with the diffraction maxima. The ratios of the experimental ring radii were then compared with those calculated from the interplanar spacings (*d*_hkl_) reported in the PDF-2 crystallographic database, enabling indexing of the diffraction pattern and identification of the crystalline phase of the synthesized material.

For Scanning Electron Microscopy-Energy-Dispersive Spectroscopy (SEM-EDS) characterization a Scios 2 DualBeam Scanning Electron Microscope with Energy-Dispersive Spectroscopy detector, Thermo Scientific, was used. The BET analysis was performed using a high-precision surface area and porosity analyzer Micromeritics Gemini VII 2390t (Norcross, GA, USA).

### 3.6. Determination of Profenofos by HPLC

To quantify profenofos, a high-performance liquid chromatography (HPLC) method was used, as described in a previous study [52]. The chromatographic separation was carried out using an RP-18 column (260 × 4 mm) obtained from Sigma Aldrich (St. Louis, MO, USA) maintained at a constant room temperature of 25 °C. The mobile phase consisted of acetonitrile:water (60:40, *v*/*v*), which remained strictly constant and unadjusted for both sample pH environments, operating under an isocratic flow rate of 2 mL/min and a 20 μL loop injection volume. Measurements were taken at 210 nm. The method was independently validated for the samples generated at pH = 4 and pH = 5. The analytical method proved to be linear, accurate, and precise under these constant parameters, exhibiting a detection limit of 0.155 mg/L and a quantification limit of 0.163 mg/L for the pH = 4 matrix, and a detection limit of 0.084 mg/L and a quantification limit of 0.130 mg/L for the pH = 5 matrix. The limits of detection and quantification were calculated using the procedure developed in a previous study [53,54].

### 3.7. Degradation Study

#### 3.7.1. Conditions for Degradation Studies

**Figure 15:**
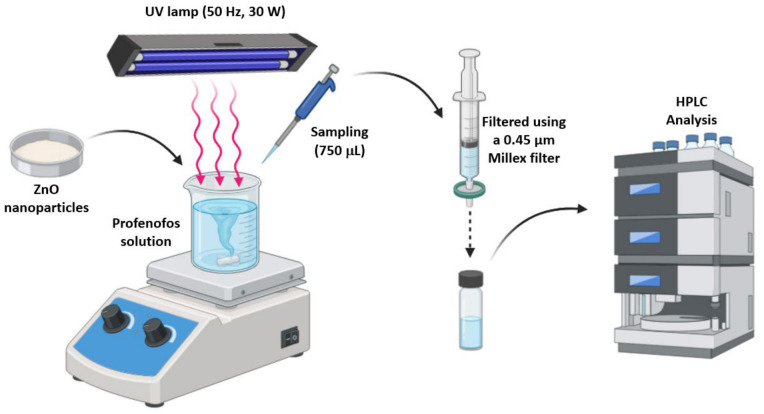
Photocatalysis system and analysis of profenofos in samples.

Aqueous solutions contaminated with profenofos (synthetic solutions) were prepared at a concentration of 20 mg/L. The solutions were prepared in 0.025% Tween and Britton-Robinson buffer at pH 4 and pH 5. A 10 mL volume of the synthetic solution was transferred to 25 mL beakers, to which the ZnO NPs were added. Subsequently, agitation was initiated under radiation using a 50 Hz, 30 W UV lamp. The agitation speed was 450 rpm for 120 min. Control groups were evaluated under identical conditions: direct photolysis was performed using UV light irradiation without adding ZnO NPs, and dark adsorption was evaluated by shielding the beaker containing the ZnO NPs from any light source. Degradation was monitored by measuring 750 μL samples, which were filtered through 0.45 μm Millex filters obtained from Sigma Aldrich (St. Louis, MO, USA) and then analyzed by HPLC (Figure 15).

#### 3.7.2. Factorial Design

To determine the pH value and ZnO NPs dosage for the profenofos degradation process, a 2^2^ × 3 factorial design was developed (Table 8). The levels for the pH factor were 4 and 5, and the levels for the ZnO NPs dosage factor were 1 and 3 g/L. The experiments were conducted in triplicate, resulting in a total of 12 experiments. The degradation experiments were conducted using a 20 mg/L profenofos solution and a stirring time of 120 min. At the end of the stirring time, the concentration of profenofos in the aqueous solutions was analyzed by HPLC. The degradation efficiency was calculated using Equation (6).

(6) $$\mathrm{Degradación}\;(\%)=\frac{C_{i}-C_{f}}{C_{i}}\times 100$$

where “C_i_” represents the initial concentration of profenofos and “C_f_” is the final concentration of profenofos or the concentration at each sampling time point. The results were analyzed using Design Expert 11 software to evaluate the factorial design and optimize the process to maximize degradation efficiency. In addition, the subsequent kinetic degradation experiments were conducted under the optimized conditions in triplicate (using independent batches of ZnO NPs) to calculate the average primary degradation efficiency.

#### 3.7.3. Degradation Kinetics

Experiments on the degradation of profenofos were conducted under optimal conditions of ZnO NPs dosage and pH. Samples were collected at 0, 1, 5, 10, 20, 30, 60, and 120 min. At the end of each time interval, the samples were analyzed by HPLC, and the adsorption capacity “q_t_” of profenofos on the ZnO NPs was calculated using Equation (7).

(7) $$q_{t}=\frac{C_{i}-C_{f}}{m}\times V$$

where “m” is the mass of ZnO NPs used in the degradation process and “V” is the volume of the profenofos solution in liters. The results were used to evaluate pseudo-first-order, pseudo-second-order, and Elovich kinetic models using Equations (8), (9) and (10), respectively.

(8) $$q_{t}=q_{e}(1-e^{-k_{1}t})$$

(9) $$\mathrm{qt}=\frac{k_{2}q_{e}^{2}t}{1+k_{2}q_{e}t}$$

(10) $$\mathrm{qt}=\frac{1}{\beta }\ln(1+\alpha \beta t)$$

where “k_1_” (min^−1^) and “k_2_” (g·mg^−1^·min^−1^) correspond to the rate constants of the pseudo-first order and pseudo-second order models, respectively. “α” and “β” are the Elovich constants, representing the initial adsorption rate (g·mg^−1^·min^−1^) and the desorption coefficient (mg·g^−1^·min^−1^), respectively. The results were analyzed using Origin Pro 2026 software.

## 4. Conclusions

ZnO nanoparticles were biosynthesized from an aqueous extract of Negra Criolla grape seeds at a concentration of 5% of the extract. The formation of ZnO nanoparticles was confirmed by UV/vis spectrophotometry, which revealed an absorption peak at 375 nm characteristic of the surface plasmon resonance of ZnO NPs. FTIR analysis revealed peaks at 872 and 703 cm^−1^ characteristic of Zn-O stretching, and the particles had an average size of 52 ± 2 nm. The biosynthesized ZnO NPs proved effective for the degradation of profenofos at an optimal dose of 3 g/L and pH = 5, achieving 59% degradation after 120 min of UV irradiation starting from an initial concentration of 20 mg/L. In conclusion, the biosynthesized ZnO NPs proved effective at degrading profenofos in synthetic solutions; however, further studies on their application in natural waters are needed to assess their potential for scale-up. Furthermore, future research should incorporate total organic carbon (TOC) analysis and liquid chromatography–tandem mass spectrometry (LC-MS/MS) to map the complete kinetics of mineralization and provide a detailed description of the degradation pathways of intermediate subproducts.

## Figures and Tables

**Table 1:** Comparison of particle sizes obtained from different aqueous extracts.

Plant Source	Type of Extract/Extraction Process	Calcination Temperature/Time	Precursor	Form	Particle Size	Source
Leaves of *Ziziphus nummularia*	Aqueous/Stirring 80 °C/1 h	400 °C/2 h	Zinc nitrate	Spherical/irregular	17.33 nm	[35]
Leaves of *Cymbopogon citratus*	Aqueous/Ultrasound 45 °C/30 min	600 °C/2 h	Zinc acetate dihydrate	Spherical	21 nm	[36]
Leaves of *Parthenium hysterophorus*	Aqueous/submersion 60 °C/15 min	400 °C/3 h	Zinc nitrate	Spherical	10 nm	[37]
Fruit of *Rhus. coriaria*	Aqueous/agitation to 750 rpm at 60 °C/2 h	---	Zinc acetate dihydrate	Spherical/hexagonal	20.51 ± 3.90 nm	[38]
Peel of *Juglans regia*	Aqueous/boil for 10 min	---	Zinc sulfate	Spherical/oval in shape	60–70 nm	[39]
Pressed “Negra Criolla” grape seed residue	Aqueous/reflux for 30 min	500 °C/2 h	Zinc acetate dihydrate	Spherical/irregular	52 ± 2 nm	This research

**Table 2:** Parameters obtained from BET analysis for the ZnO NPs biosynthesized.

Material	SSA (m^2^/g)	Total Pore Volume (cm^3^/g)	Average Pore Diameter (nm)
ZnO NPs	10.7 ± 0.7	0.0123 ± 0.0011	28 ± 1.5

**Table 3:** Percentages and degradation in the 2^2^ × 3 factorial design experiments.

N	Coded Values		Real Values		Degradation (%)
	A	B	A: Dosage (g/L)	B: pH	
1	−1	−1	1	4	15.03
2	+1	−1	3	4	52.76
3	−1	+1	1	5	27.16
4	+1	+1	3	5	59.63
5	−1	−1	1	4	16.21
6	+1	−1	3	4	51.27
7	−1	+1	1	5	28.35
8	+1	+1	3	5	60.51
9	−1	−1	1	4	14.72
10	+1	−1	3	4	53.73
11	−1	+1	1	5	26.02
12	+1	+1	3	5	59.03

**Table 4:** Analysis of variance for 2^2^ × 3 factorial design.

Source	Sum of Squares	Degrees of Freedom	Median Squares	F	*p*	Interpretation
Model	3942.42	3	1314.14	1290.24	<0.0001	Significant
A: ZnO NPs Dosage	3655.04	1	3655.04	3588.57	<0.0001	Significant
B: pH	270.66	1	270.66	265.74	<0.0001	Significant
AB	16.72	1	16.72	16.42	0.0037	Significant
Error	8.15	8	1.02			
Cor Total	3950.57	11				

**Table 5:** Values for R^2^, R^2^-adjusted, R^2^-predicted, and model precision.

Parameter	Value	Parameter	Value
Std. Dev.	1.01	R^2^	0.9979
Mean	38.7	*R*^2^-adjusted	0.9972
C.V. %	2.61	*R*^2^-predicted	0.9954
		Adequate precision	76.206

**Table 6:** Parameter values for nonlinear kinetic models.

Model	Parameter	Value
Pseudo-first-order	*k*_1_ (min^−1^)	0.166
	*q_e_*_,cal_ (mg/g)	3.530
	*R* ^2^	0.8775
Pseudo-second-order	*k*_2_ (g mg^−1^ min^−1^)	0.067
	*q_e_*_,cal_ (mg/g)	3.851
	*R* ^2^	0.9441
Elovich	*α* (mg g^−1^ min^−1^)	4.498
	*β* (g mg^−1^)	1.670
	*R* ^2^	0.9967

**Table 7:** Comparison of the degradation efficiency of profenofos using different photocatalysts.

Plant Source	Dosage (g/L)	pH	Radiation	*C_i_* (mg/L)	Time (min)	Percentage of Degradation	Reference
Fe/Ni bimetallic nanoparticles	0.3	11	UV	36.36	180	98	[46]
Silver-platinum-doped zeolite	100	6.5	UV	160	60	35	[47]
Graphitic carbon nitride/polydopamine nanocomposite	0.05	5	UV	7	25	96.4	[48]
ZnO nanoparticles	3	5	UV	20	120	59 ± 2	This study

**Table 8:** Factors and levels of factorial design.

Factor	Level	
	−1	+1
A: Dosage of ZnO NPs (g/L)	1	3
B: pH	4	5

## Data Availability

Data is contained within the article.

## Supplementary Materials

The following supporting information can be downloaded at: https://www.mdpi.com/article/10.3390/molecules31132221/s1.

## Abbreviations

The following abbreviations are used in this manuscript:

ZnO NPs	ZnO nanoparticles
GSAE	Grape seed aqueous extracts
OPPs	Organophosphorus
PF	Profenofos
ATR-FTIR	Attenuated Total Reflectance-Fourier Transform Infrared
TEM	Transmission electron microscopy
HPLC	High-resolution liquid chromatograph
XRD	X-ray diffraction
ICDD	International Centre for Diffraction Data
SAED	Selected area electron diffraction
SEM	Scanning Electron Microscopy
EDS	Energy-Dispersive Spectroscopy
BET	Brunauer–Emmett–Teller
BJH	Barrett–Joyner–Halenda
