# Supplementary material for: Photocatalytic Degradation of Profenofos Using ZnO Nanoparticles Biosynthesized with Aqueous Grape Seed Extract
Source: Molecules. 2026 Jun 24;31(13):2221. doi: 10.3390/molecules31132221 (PMC13362803; doi:10.3390/molecules31132221)
Supplement: Supplementary file 1 [file molecules-31-02221-s001.zip › molecules-4278268-supplementary.pdf]

Supplementary material

Photocatalytic Degradation of Profenofos Using ZnO Nanoparticles Biosynthesized with Aqueous Grape Seed Extract

Elvis Gilmar Gonzales-Condori[1*], Rocio Janeth Jove-Roman[2], Alfredo Quispe-Mamani[2], Gerson Márquez[1], Jeaneth Medina-Pérez[2], José Miguel Carpio-Carpio[2], Luis Lipa-Mamani[3], José A. Villanueva-Salas[2]

[1]Grupo de Investigación en Biotecnología y Ciencia de los Alimentos (GIBYCA), Universidad Tecnológica del Perú (UTP), Av. Tacna y Arica 160, Arequipa, Perú

[2]Escuela Profesional de Farmacia y Bioquímica, Universidad Católica de Santa María (UCSM), Urb. San José s/n Umacollo, Arequipa, Perú

[3]Facultad de Agronomía, Universidad Nacional de San Agustín de Arequipa, Arequipa, Perú

* Correspondence: elvgonzalesc@gmail.com; (E.G.G.-C.)

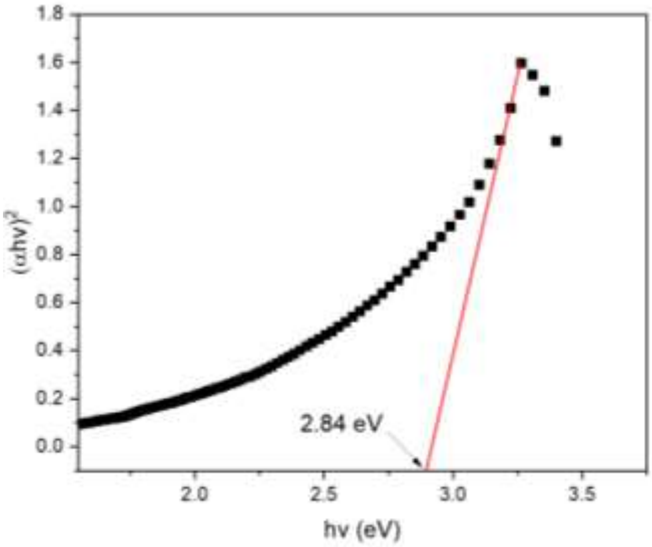

Figure S1. Bandgap energies of ZnO nanoparticles synthesized with 1 % grape seed aqueous extract determined using the Tauc method.

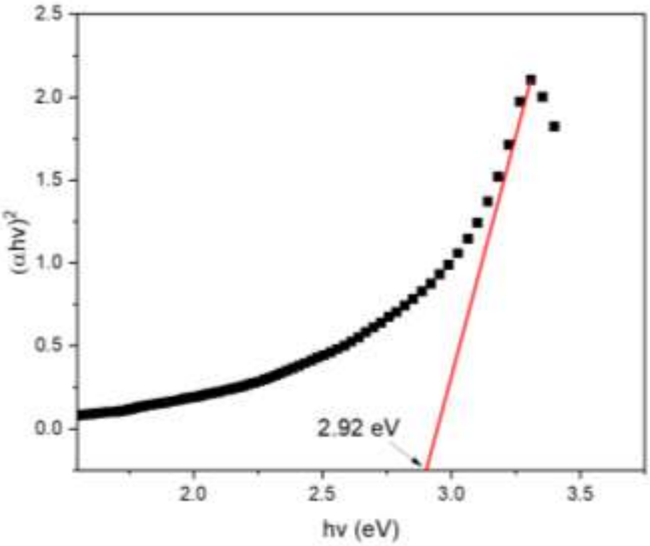

Figure S2. Bandgap energies of ZnO nanoparticles synthesized with 5 % grape seed aqueous extract determined using the Tauc method.

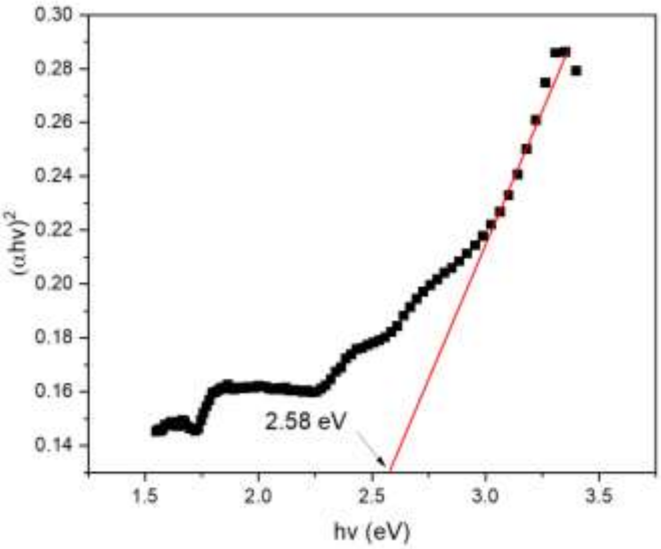

Figure S3. Bandgap energies of ZnO nanoparticles synthesized with 10 % grape seed aqueous extract determined using the Tauc method.

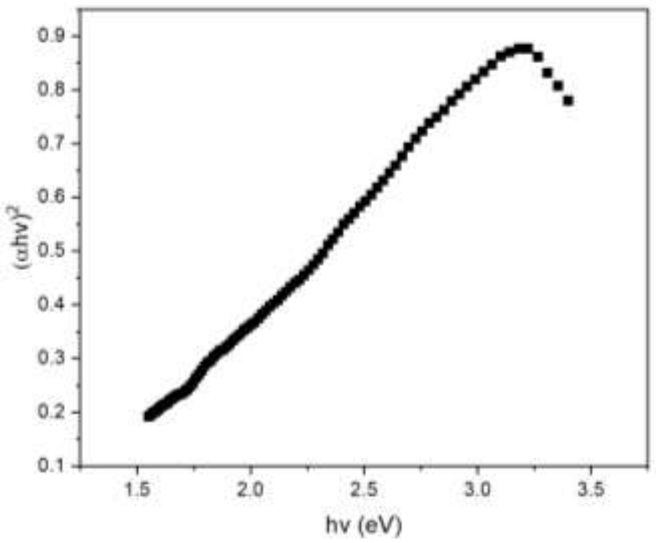

Figure S4. Tauc plot profile of ZnO nanoparticles synthesized with 15% grape seed aqueous extract (linear extrapolation not feasible due to high-concentration matrix interference).

Table S1. Values of the analysis of ZnO nanoparticles obtained using the Tauc method.

ZnO NPs	Position of the Peak / Excitatory Shoulder	Peak Energy (hν)	Eg Extrapolated
1% GSAE	385.0 nm	3.22 eV	2.84 eV
5% GSAE	375.0 nm	3.31 eV	2.92 eV
10% GSAE	370.0 nm	3.35 eV	2.58 eV
15% GSAE	385.0 nm	3.22 eV	

Note: For the 15% GSAE sample, a mathematically reliable linear extrapolation using the Tauc method could not be performed due to high baseline light scattering and absorption interference caused by the excess of biogenic organic residues; thus, the entry was omitted to ensure analytical accuracy
